# Fixed drug eruption in a cribriform pattern: an atypical presentation

**DOI:** 10.1093/skinhd/vzae006

**Published:** 2025-01-22

**Authors:** Mahesh Mathur, Neha Thakur, Sandhya Regmi, Supriya Paudel, Nabita Bhattarai, Sambidha Karki

**Affiliations:** Department of Dermatology, College of Medical Sciences Teaching Hospital, Bharatpur, Nepal; Department of Dermatology, College of Medical Sciences Teaching Hospital, Bharatpur, Nepal; Department of Dermatology, College of Medical Sciences Teaching Hospital, Bharatpur, Nepal; Department of Dermatology, College of Medical Sciences Teaching Hospital, Bharatpur, Nepal; Department of Dermatology, College of Medical Sciences Teaching Hospital, Bharatpur, Nepal; Department of Dermatology, College of Medical Sciences Teaching Hospital, Bharatpur, Nepal

## Abstract

Fixed drug eruption (FDE) is a distinct adverse drug reaction characterized by a well-defined, dusky, violaceous to erythematous patch that recurs at the same site upon re-exposure to causative drugs and resolves with hyperpigmentation. This unique reaction is a type IV hypersensitivity reaction mediated by memory CD8^+^ T cells that reside in the basal layer of the epidermis of the resting FDE lesion. Variants of FDE described in the literature include bullous, generalized bullous, nonpigmenting, linear, papular, erythema multiforme-like, transitory giant, annular, psoriasiform, erythema dyschromicum perstans-like and cellulitis-like. We present the case of a 12-year-old boy with FDE in a cribriform pattern that has not been defined so far.

## Teaser

Fixed drug eruption (FDE) is a distinct adverse drug reaction characterized by a well-defined, dusky, violaceous to erythematous patch that recurs at the same site upon re-exposure to causative drugs and resolves with hyperpigmentation. This unique reaction is a type IV hypersensitivity reaction mediated by memory CD8^+^ T-cells that reside in the basal layer of the epidermis of the resting FDE lesion. Variants of FDE described in the literature include bullous, generalized bullous, non-pigmenting, linear, papular, erythema multiforme-like, transitory giant, annular, psoriasiform, erythema dyschromicum perstans-like and cellulitis-like. We present the case of a 12-year-old male with FDE in a cribriform pattern that has not been defined so far.

**What is already known about this topic?**

- Fixed drug eruption (FDE) is characterized by same site recurrence of well-defined dusky violaceous to erythematous patch with each additional exposure to the offending agent.

**What does this study add?**

- Multiple areas of normal pigmentation within the FDE lesion showing a cribriform pattern is an unusual presentation of FDE.
- This type of unique presentation might be due to preferential infiltration of lesional epidermis by CD8+ memory T cells.

Fixed drug eruption (FDE) is a distinct type of adverse cutaneous drug eruption, first described by Bourns in 1889. The term ‘fixed drug eruption’ or ‘éruption érythémato-­pigmentée fixe’ was coined by Brocq in 1894.^1^ FDE is characterized by a well-demarcated round to oval, erythematous to violaceous patch that recurs at the same site upon re-­exposure to causative drugs in susceptible individuals and leaves postinflammatory hyperpigmentation on resolution. Common sites of involvement are the lips, anogenital area, hands and feet.^1,2^ This unique drug reaction is a type IV hypersensitivity reaction that is triggered mainly by oral medications; however, it can occur following exposure to certain foods or UVA or UVB light.^2,3^ FDE is self-limiting once the offending drug is discontinued.^1^ In this report we describe an atypical presentation of FDE in a cribriform ­pattern.

## Case report

**Figure 1:**
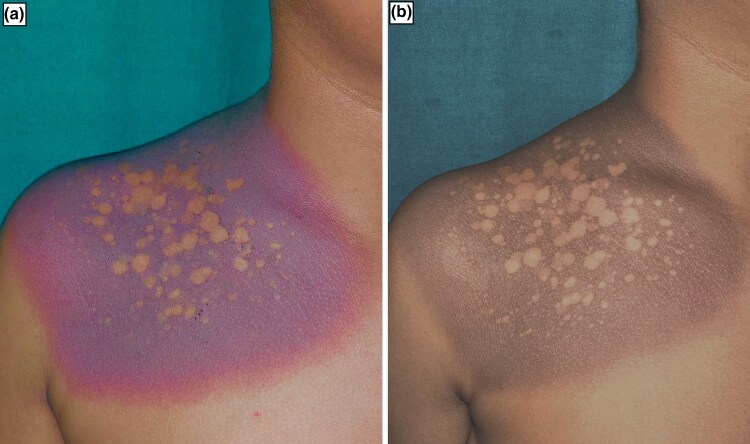
(a) Well-defined dusky, violaceous to erythematous patch with areas of normal pigmentation within the lesion over the anterior chest. (b) Postinflammatory hyperpigmentation over pre-existing lesion sparing previously uninvolved areas on follow-up.

A 12-year-old boy presented with an asymptomatic, well-­defined, dusky, violaceous to erythematous patch with multiple areas of normal pigmentation within the lesion on his chest anteriorly on the right side extending to right shoulder for 2 days (Figure 1a). He took paracetamol (acetaminophen) for fever, after which the lesion developed. There was a history of a similar lesion 2 years previously at the same site on the chest after intake of over-the-counter medication, which subsided gradually. Mucosae examination revealed normal findings.

**Figure 2:**
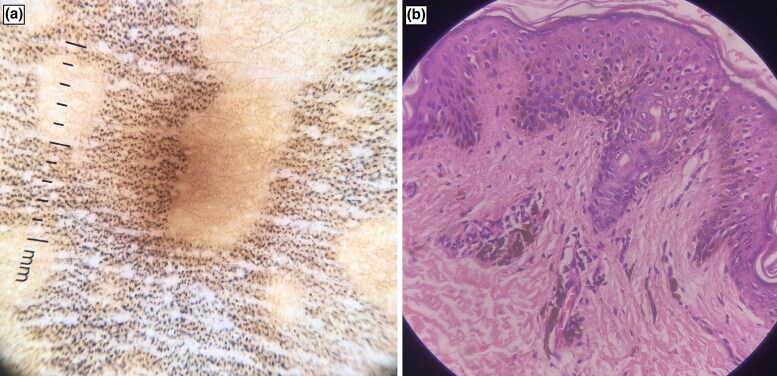
(a) Dermoscopy (DL4 polarized, ×10) revealed multiple brown and grey dots grouped in a pattern with islands of sparing showing a normal reticular pigmented network. (b) Haematoxylin and eosin staining (×40) showed basal cell vacuolization, melanin incontinence and perivascular inflammatory infiltrates.

Routine blood investigations performed were normal except for eosinophilia. Dermoscopy revealed multiple brown and grey dots grouped in a pattern with islands of sparing showing a normal reticular pigmented network (Figure 2a). Skin biopsy showed basal cell vacuolization, melanin incontinence in the upper dermis and perivascular inflammatory infiltrates (Figure 2b). The Naranjo adverse drug reaction probability scale value was calculated to be 7, which indicates a probable adverse drug reaction to paracetamol (acetaminophen). Patch testing was not done as it was not available at our centre. Based on clinical, dermoscopic and histological findings, a diagnosis of FDE was made and the patient was prescribed topical steroid twice daily. The patient was advised to avoid triggering medication. He presented after 1 month for follow-up and on examination hyperpigmentation was present over the pre-existing lesion with sparing of prior uninvolved areas (Figure 1b).

## Discussion

FDE is characterized by same-site recurrence of the lesion and can increase in size and number with additional exposure to the offending medication.^2,3^ The mechanisms responsible for preferential localization of FDE lesions to certain skin sites are poorly understood. However, evidence suggests that intraepidermal CD8^+^ T cells in the lesion are the final effector cells that lead to selective destruction of the lesional epidermis. Spontaneous resolution on withdrawal of the triggering agent occurs due to infiltration of the lesional epidermis by regulatory CD4^+^ T cells. After clinical resolution, effector-memory CD8^+^ T cells with epidermal localization capacity persist in the basal layer of the epidermis of the resting FDE lesion.^4^ Several morphological variants of FDE described in the literature are bullous, generalized bullous, nonpigmenting, linear, papular, erythema multiforme-like, transitory giant, annular, psoriasiform, DL4 polarized-like and cellulitis-like.^5^ Patchy islands of sparing in the active as well as the pigmented FDE lesion, as seen in our case, might be due to preferential infiltration of lesional epidermis by CD8^+^ memory T cells, which is an unusual presentation for FDE. FDE presenting in such a cribriform pattern has not been described in the literature so far.

FDE can be diagnosed on clinical grounds; however, skin biopsy, topical patch test and systemic rechallenge may help to confirm the diagnosis.^1,2^ Histopathologically, FDE is characterized by individual necrotic keratinocytes, vacuolar interface dermatitis, perivascular inflammatory infiltrates and pigment incontinence, as seen in our case.^2,3^ Dermoscopy further aids the diagnosis. Differential diagnoses include erythema multiforme, contact dermatitis, cellulitis and herpes simplex infection, which were excluded by history, clinical presentation and histopathology. Treatment is mainly symptomatic with topical or systemic corticosteroids and antihistamines, and avoidance of the offending drug is of paramount importance.^1,3^

Being a self-resolving condition, reassurance and avoidance of the culprit drug is of utmost importance in FDE. It is important to differentiate FDE from common clinical mimics. To the best of our knowledge, FDE presenting in a cribriform pattern, as seen in our case, has not been reported so far in the literature. Dermoscopic features are remarkable, and histopathological changes describing almost all findings are reported in previous literature. We hope this case report will make clinicians familiar with an atypical presentation of FDE.

## Data Availability

The data that support the findings of this study are available from the corresponding author upon reasonable request.
